# Mailed Audit and Feedback for Antibiotic Prescribing in Primary Care

**DOI:** 10.1001/jamanetworkopen.2026.1641

**Published:** 2026-03-13

**Authors:** Lisa Masucci, Kevin Schwartz, Noah Ivers, Li Bai, Kevin Brown, Mina Tadrous, Jeremy M. Grimshaw, Holly O. Witteman, Lindsay Friedman, Bradley J. Langford, Valerie Leung, Tara Gomes, Gary Garber, Monica Taljaard, Jennifer Shuldiner, Sharon Gushue, Michael Silverman, Nick Daneman, Jamie Brehaut, Justin Presseau, Jerome A. Leis, Meagan Lacroix, Merrick Zwarenstein, Kednapa Thavorn

**Affiliations:** 1 Ottawa Hospital Research Institute, The Ottawa Hospital, Ottawa, Ontario, Canada; 2 Public Health Ontario, Toronto, Canada; 3 ICES, Toronto, Ontario, Canada; 4 Li Ka Shing Knowledge Institute, Unity Health Toronto, Toronto, Ontario, Canada; 5 Dalla Lana School of Public Health, University of Toronto, Toronto, Ontario, Canada; 6 Women’s College Hospital Research Institute, Women’s College Hospital, Toronto, Ontario, Canada; 7 Leslie Dan Faculty of Pharmacy, University of Toronto, Toronto, Ontario, Canada; 8 Department of Medicine, University of Ottawa, Ottawa, Ontario, Canada; 9 Department of Family and Emergency Medicine, Laval University, Quebec City, Quebec, Canada; 10 Michael Garron Hospital, Toronto East Health Network, Toronto, Ontario Canada; 11 Institute of Health Policy, Management and Evaluation, University of Toronto, Toronto, Ontario, Canada; 12 Department of Family and Community Medicine, University of Toronto, Toronto, Ontario, Canada; 13 Ontario Health, Toronto, Ontario, Canada; 14 Western University, London, Ontario, Canada; 15 Sunnybrook Health Sciences Centre, University of Toronto, Toronto, Ontario, Canada; 16 Sunnybrook Research Institute, Sunnybrook Health Sciences Centre, University of Toronto, Toronto, Ontario, Canada; 17 Department of Family Medicine, Epidemiology & Biostatistics, Schulich School of Medicine and Dentistry, Western University, London, Ontario, Canada

## Abstract

**Importance:**

Inappropriate antimicrobial use is a major but modifiable contributor to antimicrobial costs and harms. To address this global threat, many countries have implemented national stewardship programs. However, the cost-effectiveness of such programs targeting primary care physicians remains unknown.

**Objective:**

To determine the economic value of an antibiotic audit and feedback (A&F) program from a Canadian public payer perspective.

**Design, Setting, and Participants:**

This economic evaluation used data from a randomized clinical trial of primary care physicians in Ontario, Canada, in which a mailed A&F intervention reduced antibiotic prescribing among patients 65 years and older. Trial data were linked with administrative databases to estimate the program costs and savings from reduced antibiotic use, adverse events, and undertreatment harms. A decision model was developed to calculate monetary costs and benefits. Data were collected from January 1 to July 1, 2022, and were analyzed from March 1 to April 1, 2025. All costs are reported in 2024 Canadian dollars.

**Intervention:**

A mailed A&F letter sent to primary care physicians with their antibiotic prescribing rate compared with their peers, with additional messaging about the harms of unnecessary antibiotics.

**Main Outcomes and Measures:**

Net costs and benefits, return on investment (ROI), and probability of achieving a positive ROI (>1). Uncertainty was explored using probabilistic analysis with 5000 simulations and deterministic sensitivity analyses.

**Results:**

The study included 4879 primary care physicians, of whom 2711 (55.6%) were male, with a mean (SD) of 25.0 (13.3) years since medical school graduation. The program cost was $5.50 per physician and generated $43.03 in savings. The ROI was $8.82 (95% uncertainty interval, $1.32-$22.56) per dollar invested. The probability that the antibiotic A&F program provided an ROI greater than 1 was 93.2%. Scaling the program to all primary care physicians in Ontario would further improve the estimated ROI. Results were robust across sensitivity analyses.

**Conclusions and Relevance:**

In this economic evaluation of a mailed antibiotic A&F intervention, the intervention was associated with substantial economic and clinical value. These findings support A&F as a scalable, low-cost component of antimicrobial stewardship programs and highlight its potential to optimize prescribing and reduce antimicrobial resistance.

## Key Points

**Question:**

What is the economic value of an antibiotic audit and feedback program targeting primary care physicians?

**Findings:**

In this economic evaluation using data from a randomized clinical trial of 4879 physicians in Ontario, Canada, every dollar invested in the mailed antibiotic audit and feedback program generated an estimated return of $8.82 in Canadian dollars.

**Meaning:**

These findings suggest that a low-cost, scalable audit and feedback intervention can deliver substantial economic and clinical value, supporting its adoption as a high-value antimicrobial stewardship strategy in primary care.

## Teaser

This economic evaluation estimates the return on investment of an audit and feedback program for antibiotic prescribing from a Canadian public health care payer perspective.

## Introduction

In 2021, it was estimated that worldwide, 1.14 million people died of antimicrobial-resistant infections.^1^ These infections contribute to considerable health care costs, estimated to be as high as USD $3.5 billion between 2015 and 2050 per year for 33 Organisation for Economic Co-operation and Development countries.^2,3^ For the years 2015 to 2050, antimicrobial resistance is expected to cost USD $60 billion to European Union health systems and USD $74 billion to those of Canada, the US, and Australia combined.^2^

The misuse of antimicrobials in health care is a key modifiable driver of antimicrobial resistance.^4^ Previous research suggests that at least one-quarter of antibiotics prescribed in primary care settings are unnecessary.^5,6^ To address this issue, antimicrobial stewardship programs have been implemented in some regions to support health care professionals to follow evidence-based prescription guidelines.^4^ One commonly used strategy is audit and feedback (A&F), which involves comparing clinicians’ prescribing practices against the performance of peers or benchmarks to promote improved clinical decision-making.^7^ A systematic review of 56 randomized trials from 16 countries^8^ showed that A&F can effectively reduce antibiotic prescribing in primary care; however, there is limited evidence on the cost-effectiveness of these interventions in reducing overall antibiotic use.^9^ While evidence on the effectiveness of audit and feedback interventions is growing, policymakers and stewardship teams require accumulating economic evidence to support sustained investment and broader implementation of these A&F programs.

A randomized clinical trial (RCT) of physicians^10^ was previously conducted to evaluate an antibiotic A&F program. The program involved mailing physicians a letter containing individualized antibiotic prescribing data for patients 65 years and older compared with no letter.^10^ The trial demonstrated a significant relative reduction of 5% in overall antibiotic prescribing (relative rate, 0.95; 95% CI, 0.94-0.96). Based on the findings of the RCT, we estimated the return on investment (ROI) of the A&F program for antibiotic prescribing from the perspective of a Canadian public health care payer.

## Methods

### Study Design

This economic evaluation received ethics research board approvals from Women’s College Hospital Research Institute and Public Health Ontario, Toronto, Ontario, Canada, who approved a waiver of informed consent for trial enrollment. The study is reported in accordance with the Consolidated Health Economic Evaluation Reporting Standards (CHEERS) reporting guideline.

A cost-benefit analysis was conducted based on data from an RCT that randomized 4879 physicians in Ontario to receive either an antibiotic A&F program intervention or no intervention (control group).^10^ The A&F program consisted of a mailed letter that reported each physician’s antibiotic prescribing rate compared with their peers. The trial used a 2-by-2 factorial trial design to also assess the added effects of (1) a harms messaging infographic and (2) prescribing data adjusted for patient and practice characteristics.^10^ The trial focused on community-dwelling patients 65 years and older, as prescription data for this group were available through administrative databases, which is considered an accurate source for medication use data.^11^ The trial outcomes, as well as this economic evaluation, used administrative databases housed and analyzed at ICES (formerly the Institute for Clinical Evaluative Sciences), where there was no loss to follow-up.^10^

A decision-analytic model was developed to estimate the net benefits of each intervention strategy evaluated in the trial. The model captured key clinical pathways by simulating the probability of antibiotic prescribing vs no antibiotic prescribing for each strategy. For those patients who were prescribed antibiotics, the model incorporated the probability of experiencing antibiotic-related adverse events (Table 1). For those who were not prescribed antibiotics, the probability of experiencing harms due to undertreatment was modeled. The frequency of patients experiencing any adverse event or any undertreatment harm was used to inform the parameter estimates. Model parameters were derived from the RCT^10^ and linked health care utilization and cost data from ICES. ICES is designated as a prescribed entity in that it is permitted to hold an inventory of coded and linkable health datasets for much of the publicly funded administrative health services records for the Ontario population under the province’s Personal Health Information Protection Act.

**Table 1.** Input Parameters

Parameter	Parameter estimate (SE)^a^	Distribution	Source
Costs per physician			
Program cost per physician	$5.50 ($0.70)	Gamma	A&F program team
Antibiotic prescription cost	$553.00 ($8.38)	Gamma	ODB database
Adverse event cost	$13 745.00 ($488.00)	Gamma	CIHI and OHIP databases
Undertreatment harms cost	$77 961.00 ($2116.00)	Gamma	CIHI database
Probabilities			
Proportion with antibiotic prescription: control	0.059 (0.00067)	Beta	Schwartz et al,^10^ 2024
Proportion with antibiotic prescription: antibiotic A&F program	0.056 (0.00063)	Beta	Schwartz et al,^10^ 2024
Received antibiotics			
Proportion with an adverse event: control	0.103 (0.013)	Beta	CIHI and OHIP databases
Proportion with an adverse event: antibiotic A&F program	0.105 (0.013)	Beta	CIHI and OHIP databases
No antibiotics			
Proportion with undertreatment harms: control	0.026 (0.003)	Beta	CIHI database
Proportion with undertreatment harms: antibiotic A&F program	0.026 (0.003)	Beta	CIHI database
Relative rate of receiving an antibiotic prescription for the A&F program vs usual care	0.95 (0.005)	Lognormal	Schwartz et al,^10^ 2024
Relative rate of having an adverse event for the A&F program vs usual care	0.98 (0.021)	Lognormal	CIHI and OHIP databases
Relative rate of undertreatment harms for the A&F program vs usual care	0.98 (0.016)	Lognormal	CIHI database

Abbreviations: A&F, audit and feedback; CIHI, Canadian Institute for Health Information; ODB, Ontario Drug Benefit; OHIP, Ontario Health Insurance Program.

^a^ All costs are reported in Canadian dollars.

A 6-month time horizon was adopted to align with the primary outcome of the trial—overall antibiotic prescribing—measured during the 6 months following program implementation, from January 1 to July 1, 2022. This time frame reflects the intended duration of the intervention and captures the relevant costs and benefits associated with the A&F program during the study period. Costs included program implementation expenses, while benefits captured reductions in antibiotic costs and downstream health care costs related to adverse events (eg, diarrhea and thrush) or bacterial infection complications from potential undertreatment (eg, pneumonia and pericarditis). The ROI was estimated by subtracting incremental benefits from incremental costs and dividing by the incremental costs. All costs were estimated in 2024 Canadian dollars and are reported as such.

### Program Costs

The costs associated with the antibiotic A&F program are reported in the input parameters in Table 1. The A&F program costs included personnel time for coding and customizing letters based on each physician’s prescribing patterns. Additional personnel time was allocated for program management activities required for ensuring the accuracy of reports and coordinating mailing letters. We also included per-physician costs for printing and mailing individualized letters based on individual antibiotic prescribing data. This intervention did not require the development of a database to deliver the program, as it relied on existing administrative records.

### Economic Costs

The benefits were quantified as the monetary value of antibiotic prescriptions avoided, adverse events avoided, and potential undertreatment harms avoided (assessed by hospitalizations for severe bacterial infections) attributed to the intervention. These clinical outcomes were not valued using the willingness-to-pay approach. To estimate these economic costs, we used trial data to derive the probability of receiving an antibiotic prescription in both the antibiotic A&F program and control groups.^10^ The analysis was restricted to family physicians who had patient encounters during the 6-month trial period, excluding the top 1% of prescribers based on their baseline antibiotic prescribing rates. This economic evaluation was conducted leveraging an RCT among family physicians; therefore, the study population and setting reflect the eligibility criteria and design of the original trial. These physicians were linked to administrative data, from which we identified antibiotic use, adverse events associated with antibiotic use, and potential bacterial infection complications—used as a proxy for potential harms from undertreatment. A detailed list of antibiotics, adverse events, and undertreatment harms is provided in eTable 1 in Supplement 1.

Costs that informed our model were directly measured using individual-level linked administrative health data from the Ontario Health Insurance Plan and Canadian Institute for Health Information Databases housed at ICES (eTable 2 in Supplement 1). Physician visits were identified using the Ontario Health Insurance Plan Claims Database; emergency department visits and same-day surgery, using the National Ambulatory Care Reporting System; inpatient hospitalizations, using the Discharge Abstract Database; adult inpatient rehabilitation, using the National Rehabilitation Reporting System; hospital-based and long-term care services, using the Complex Continuing Reporting System; prescription drug costs, using the Ontario Drug Benefit database; hospital-based and long-term care services, using the Complex Continuing Care Reporting System; and laboratory and diagnostic tests, using the Ontario Laboratory Information System. For antibiotics, we obtained the costs using the drug identification number of 26 antibiotics (eTable 1 in Supplement 1). Costs of adverse events included hospitalizations and ambulatory visits resulting from drug-related adverse events. Undertreatment harms also included hospitalizations resulting from ear, nose, and throat infections; skin and soft tissue infections; invasive infections; and rhematic fever identified using prespecified codes. For each participant, costs across all relevant encounters during the follow-up period were summed to generate total costs, and mean per-patient costs were calculated for the A&F program and control group.

### Statistical Analysis

Data were analyzed from March 1 to April 1, 2025. To estimate the differences in overall antibiotic prescription, adverse events, and undertreatment harms costs between groups, we used a generalized linear model with adjustment for the following covariates: baseline costs, years since physician graduation, physician sex, and a stratification variable indicating whether a physician had previously participated in an earlier antibiotic A&F trial.^12^ We used a similar analytic approach to the original RCT and adjusted for the same prespecified covariates from this trial, which were initially selected to optimize statistical power. Where appropriate, we assessed statistically significant reductions indicated using 2-sided *P* < .05.

The ROI was calculated by subtracting the incremental monetary costs (costs of A&F program minus costs of usual care) from the incremental monetary benefits (A&F program benefits minus usual care benefits) and dividing the result by the incremental monetary costs (costs of A&F program minus costs of usual care).^13^ An ROI greater than 1 indicated that the program’s savings exceeded its costs, whereas an ROI less than 1 indicated that the program was not cost reducing. We conducted deterministic and probabilistic sensitivity analyses to assess the robustness of the study results. For the base case, a probabilistic analysis was performed to account for uncertainty across all parameters. Parameters were sampled from appropriate distributions, which were informed by the plausible ranges (Table 1). Specifically, beta distributions were used for probabilities and gamma distribution for costs. We performed 5000 simulations per group to estimate the mean outcomes and calculated the probability that the antibiotic A&F program would yield a positive return on investment (ie, ROI >1). Uncertainty intervals (UIs) were calculated to quantify the range of possible values around the ROI.

Deterministic 1-way sensitivity analyses were conducted to assess how variation in individual parameters were associated with the results, including (1) antibiotic A&F program costs, (2) the relative rate of receiving an antibiotic prescription, (3) the relative rate of having an adverse event, and (4) the relative rate of undertreatment harms. We also performed a scenario analysis to assess how scaling to all family physicians in Canada (approximately 40 000) would be associated with the ROI. This scenario analysis assumed that the per-physician cost of printing and mailing the antibiotic A&F reports remained constant, while personnel and program management costs associated with tailoring data, ensuring report accuracy, and coordinating mailings would decrease as the program scaled to include more physicians.

## Results

**Figure 1.**
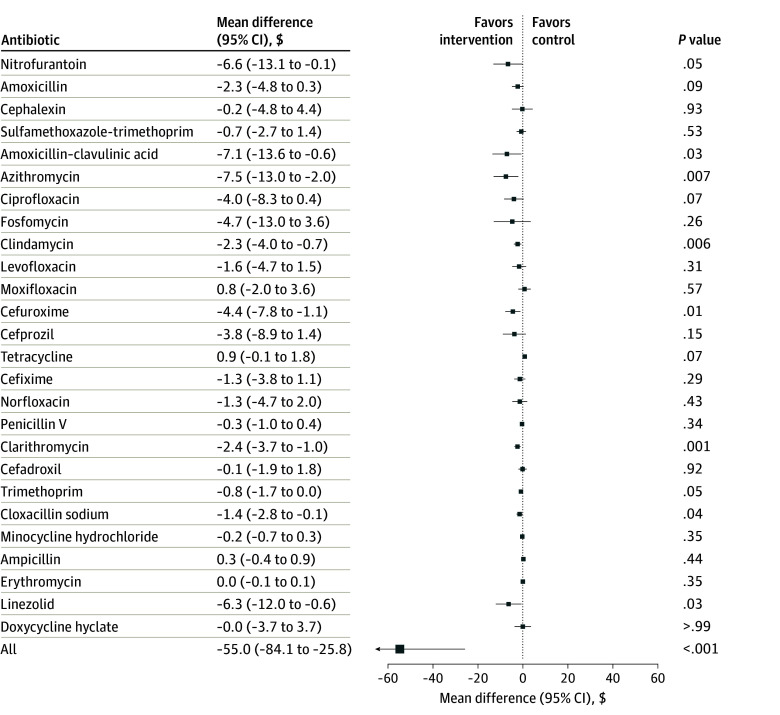
Forest Plot Showing Mean Difference in Costs for Antibiotic Use Costs are reported in Canadian dollars.

**Figure 2.**
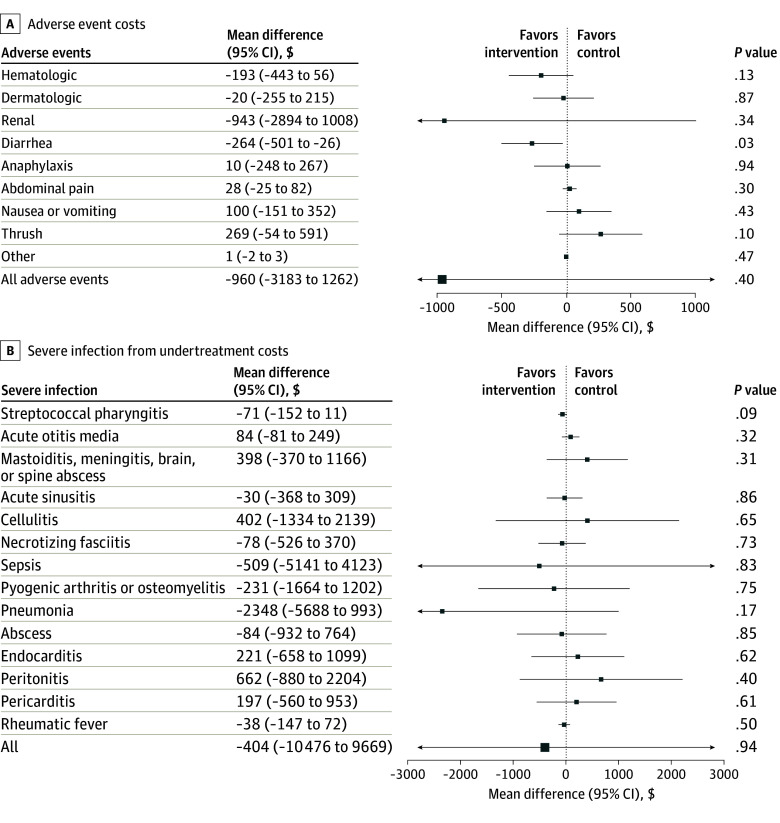
Forest Plots Showing Mean Differences in Costs Due to Adverse Events and Undertreatment Costs are reported in Canadian dollars.

This study included 4879 primary care physicians, 3909 in the intervention group and 970 in the control group. A total 2168 physicians (44.4%) were female and 2711 (55.6%) were male. Mean (SD) time since graduation was 25.0 (13.3) years. The estimated cost per physician to deliver the antibiotic A&F program was $5.50 (SE, $0.70). This cost included a fixed $3.62 per physician for data acquisition, analysis, quality assurance, and program management, and a variable cost of $1.88 for printing, postage, and personnel to mail the letter. During the 6-month period, the program resulted in lower government spending on antibiotic prescriptions—$520.00 per physician in the intervention group compared with $586.00 in the control group. As shown in Figure 1, this represents a statistically significant reduction of $55.00 (95% CI, −$84.10 to −$25.80) in antibiotic costs per physician. On average, the program reduced 3 antibiotic prescriptions per 1000 patients. Notable cost reductions were observed for several prescribed antibiotics, including azithromycin, amoxicillin–clavulanic acid, nitrofurantoin, linezolid, cefuroxime, clarithromycin, clindamycin, and cloxacillin. The A&F program also resulted in a nonsignificant decrease in health care utilization costs related to adverse events (mean difference, −$960.00; 95% CI, −$3183.00 to $1262.00). A statistically significant reduction in costs was observed for diarrhea (mean difference, −$264.00; 95% CI, −$501.00 to −$26.00) (Figure 2A). Although the program reduced the undertreatment-related costs by $404.00 (95% CI, −$10 476.00 to $9669.00) per physician, this result was not statistically significant (Figure 2B).

**Figure 3.**
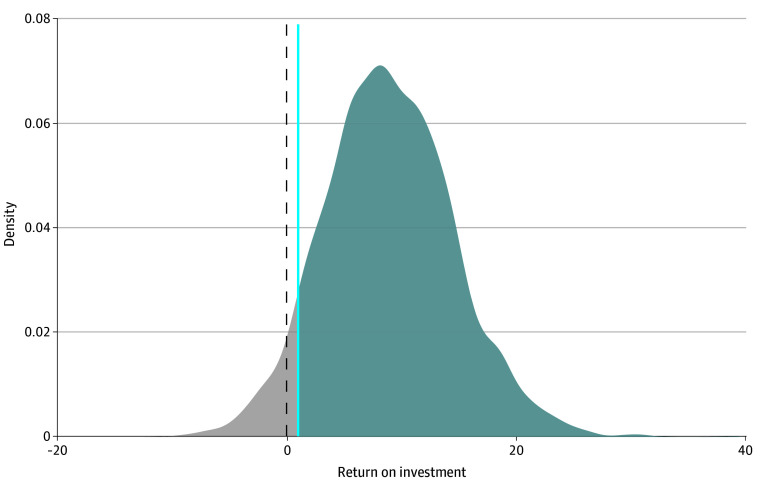
Density Plot of Return on Investment Distribution The probability of a positive return on investment was 93.2%.

The total monetary savings were $43.03 per physician and the ROI was $8.82 (95% UI, $1.32-$22.56) per dollar spent. The probability of the antibiotic A&F program being a positive ROI (>1) was 93.2% (Figure 3). Sensitivity analyses showed that the results were generally robust across parameter ranges (Table 2). Scenario analyses demonstrated increasing returns with scale. Specifically, enrolling 40 000 primary care physicians (all family physicians in Canada) would increase the estimated ROI to $16.82 (95% UI, $4.10-$36.91) for every dollar invested (eFigure in Supplement 1).

**Table 2.** Deterministic Sensitivity Analysis^a^

Parameter	Difference		ROI
	Benefits (savings)	Total cost	
Base case results	$43.03	$5.50	$8.82
Antibiotic A&F program cost per physician: $4.27	$42.69	$4.40	$10.71
Antibiotic A&F program cost per physician: $6.41	$43.40	$6.60	$7.57
Relative rate of receiving antibiotic prescription for the A&F program vs usual care: 0.94	$43.54	$5.50	$8.91
Relative rate of receiving antibiotic prescription for the A&F program vs usual care: 0.96	$43.94	$5.50	$8.98
Relative rate of having an adverse event for the A&F program vs usual care: 0.94	$43.60	$5.50	$8.93
Relative rate of having an adverse event for the A&F program vs usual care: 1.02	$42.77	$5.50	$8.78
Relative rate of undertreatment harms for the A&F program vs usual care: 0.95	$43.02	$5.50	$8.82
Relative rate of undertreatment harms for the A&F program vs usual care: 1.01	$42.99	$5.52	$8.79

Abbreviations: A&F, audit and feedback; ROI, return on investment.

^a^ All costs are reported in Canadian dollars.

## Discussion

This study highlights the economic value of implementing a mail-based antibiotic A&F program for family physicians. From the perspective of a public payer, every dollar invested in the program generated a return of $8.82. We assessed the ROI using data from a large RCT that captures the effectiveness of interventions in routine clinical settings while increasing the generalizability of our findings to broader health care systems.

Three systemic reviews^9,14,15^ have summarized the economic evaluation of antimicrobial stewardship programs and educational interventions in primary care. Across these reviews, A&F interventions consistently demonstrated good value for money, with some programs found to be cost-saving (ie, dominant), and others reporting up to €105.43 (US $130.16) per percentage increase in appropriate first-line prescribing for urinary tract infections.^16,17,18,19,20^ Differences may reflect differently designed A&F interventions and may also have to do with baseline prescribing patterns. In the case of our program, because approximately 25% of antibiotic prescribing prior to the program was deemed unnecessary, there was considerable room for improvements.^5^

One economic evaluation^19^ used data from an RCT in which physicians participated in monthly peer review meetings focused on antibiotic prescribing among children with upper respiratory infections. That study reported an incremental cost of USD $0.03 per percentage point reduction in antibiotic prescribing.^19^ Three other studies^16,17,18^ evaluated feedback interventions through randomized designs and similarly found A&F to be more cost-effective than usual care.

An economic evaluation conducted in British Columbia, Canada,^20^ used a time-series analysis to assess a population-based education program targeting prescribers, pharmacists, and the public. The program demonstrated a strong ROI, saving $76.20 for every dollar spent.^20^ However, unlike our RCT-based approach, the observational design limited causal inference. Additionally, the broader scope of that intervention—which included both system-wide and patient-level costs—may have contributed to its higher observed returns.

Our findings underscore both the economic and clinical value of an antimicrobial A&F program in primary care. The program led to reductions in antibiotic use and diarrhea and was demonstrated to be cost-effective. These findings may inform policy decisions to invest in and expand community-based antimicrobial stewardship initiatives that feature A&F. Future research should examine the sustainability and long-term cost-effectiveness of A&F programs across diverse health care systems to inform broader implementation.

### Limitations

This study has several limitations. First, we used the overall reduction in antibiotic prescribing to derive the program savings because administrative data did not permit assessment of the appropriateness of each prescription. While previous research has shown that approximately 25% of antibiotics prescribed by primary care physicians in Ontario are unnecessary,^5^ the absence of direct measures of prescription appropriateness may have limited our ability to fully capture the program’s clinical impact. However, we evaluated potential harms from undertreatment, and no signal for this was identified. Second, the 95% CIs around incremental cost estimates for antibiotics, adverse events, and undertreatment harms were wide. This is not unexpected, as health care cost data are typically highly skewed. To address this, we conducted a probabilistic analysis that incorporated parameter uncertainty using appropriate statistical distributions. Third, our findings are specific to this particular antibiotic A&F program and may not be generalizable to other A&F programs, which can vary in design, implementation, resource requirements, and associated costs. We relied on existing administrative databases to deliver the intervention; therefore, the costs of developing a program-specific database were not included in the overall program cost. As such, implementation costs may differ in settings where prescribing or dispensing databases are not routinely available. Nonetheless, our scenario analyses suggested that the ROI estimates were robust to modifications in program delivery. Similarly, the costs of antibiotics and health care services are context dependent and may differ across health care systems and jurisdictions. Fourth, the trial population was limited to patients 65 years and older, which may restrict the generalizability to younger populations. This limitation may lead to an underestimation of the broader population-level savings, as Saqib et al^21^ have previously demonstrated that this intervention had spillover effects to younger groups. Fifth, we only considered a 6-month time frame and did not assume sustained or cumulative effects beyond the time horizon. If reductions in prescribing were to continue after 6 months, the cost-savings may be even greater. Conversely, if the impact of the intervention were to wane over time, then the ROI would be reduced. The long-term impact of a 1-time audit and feedback intervention is therefore uncertain and may depend on whether the effects are reinforced through repeated implementation.^10^ Finally, our analysis focused on direct and immediate cost impacts associated with changes in antibiotic prescribing and related adverse events. We did not model broader population-level externalities arising from reduced antimicrobial resistance, which may accrue over longer time horizons and extend beyond the health care system. Although quantifying these effects is methodologically challenging, reductions in inappropriate antibiotic use are likely to generate additional downstream health and economic benefits. As a result, our findings likely underestimate the full societal value of audit and feedback interventions targeting antibiotic stewardship.

## Conclusions

In this economic evaluation of a mailed antibiotic A&F intervention, we found that the intervention offered substantial economic and clinical value. Our findings provide compelling evidence for health care decision-makers on the economic and clinical benefits of implementing an A&F program in primary care. The ROI increases with broader participation, indicating even greater economic savings at scale. Most savings were driven by reductions in adverse events linked to unnecessary antibiotic use, highlighting both the economic and patient safety benefits of the A&F program. These findings support the integration of antibiotic A&F interventions into routine primary care as a scalable strategy to advance antimicrobial stewardship, improve prescribing practices, and enhance the sustainability of health care delivery.

## Notes

Supplement 1. **eTable 1.** List of Adverse Events, Undertreatment Harms, and Antibiotics **eTable 2.** Data Sources **eFigure.** Return on Investment by Number of Family Physicians

Supplement 2.
Data Sharing Statement

## References

[1] Collaborators GBDAR; GBD 2021 Antimicrobial Resistance Collaborators. Global burden of bacterial antimicrobial resistance 1990-2021: a systematic analysis with forecasts to 2050. Lancet. 2024;404(10459):1199-1226. doi:10.1016/S0140-6736(24)01867-1 39299261 PMC11718157

[2] Stemming the superbug tide: just a few dollars more. OECD Health Policy Studies. November 7, 2018. Accessed June 1, 2025. https://www.oecd.org/en/publications/stemming-the-superbug-tide_9789264307599-en.html

[3] Thorpe KE, Joski P, Johnston KJ. Antibiotic-resistant infection treatment costs have doubled since 2002, now exceeding $2 billion annually. Health Aff (Millwood). 2018;37(4):662-669. doi:10.1377/hlthaff.2017.1153 29561692

[4] Using audit and feedback for health professionals to improve the quality and safety of health care. World Health Organization. October 1, 2010. Accessed June 1, 2025. https://www.who.int/europe/publications/i/item/2077-1584

[5] Schwartz KL, Langford BJ, Daneman N, . Unnecessary antibiotic prescribing in a Canadian primary care setting: a descriptive analysis using routinely collected electronic medical record data. CMAJ Open. 2020;8(2):E360-E369. doi:10.9778/cmajo.20190175 32381687 PMC7207032

[6] Silverman M, Povitz M, Sontrop JM, Shariff SZ. Antibiotic prescribing for nonbacterial acute upper respiratory infections in elderly persons. Ann Intern Med. 2017;167(10):758-759. doi:10.7326/L17-0438 29159387

[7] Busse R, Klazinga N, Panteli D, . Improving Healthcare Quality in Europe: Characteristics, Effectiveness and Implementation of Different Strategies. European Observatory on Health Systems and Policies; 2019. 31721544

[8] Xu AXT, Brown K, Schwartz KL, . Audit and feedback interventions for antibiotic prescribing in primary care: a systematic review and meta-analysis. Clin Infect Dis. 2025;80(2):253-262. doi:10.1093/cid/ciae604 39657007 PMC11848270

[9] Moore L, Guertin JR, Tardif PA, . Economic evaluations of audit and feedback interventions: a systematic review. BMJ Qual Saf. 2022;31(10):754-767. doi:10.1136/bmjqs-2022-014727 35750494

[10] Schwartz KL, Shuldiner J, Langford BJ, . Mailed feedback to primary care physicians on antibiotic prescribing for patients aged 65 years and older: pragmatic, factorial randomised controlled trial. BMJ. 2024;385:e079329. doi:10.1136/bmj-2024-079329 38839101 PMC11151833

[11] Levy AR, O’Brien BJ, Sellors C, Grootendorst P, Willison D. Coding accuracy of administrative drug claims in the Ontario Drug Benefit database. Can J Clin Pharmacol. 2003;10(2):67-71. 12879144

[12] Schwartz KL, Ivers N, Langford BJ, . Effect of antibiotic-prescribing feedback to high-volume primary care physicians on number of antibiotic prescriptions: a randomized clinical trial. JAMA Intern Med. 2021;181(9):1165-1173. doi:10.1001/jamainternmed.2021.2790 34228086 PMC8261687

[13] Pokhrel S, Owen L, Coyle K, Coyle D. ROI in Public Health Policy: Supporting Decision-making. Palgrave Pivot Cham; 2017. doi:10.1007/978-3-319-68897-8

[14] Rocha V, Estrela M, Neto V, Roque F, Figueiras A, Herdeiro MT. Educational interventions to reduce prescription and dispensing of antibiotics in primary care: a systematic review of economic impact. Antibiotics (Basel). 2022;11(9):11. doi:10.3390/antibiotics11091186 36139965 PMC9495011

[15] Wubishet BL, Merlo G, Ghahreman-Falconer N, Hall L, Comans T. Economic evaluation of antimicrobial stewardship in primary care: a systematic review and quality assessment. J Antimicrob Chemother. 2022;77(9):2373-2388. doi:10.1093/jac/dkac185 35724206 PMC9410674

[16] Gillespie P, Callan A, O’Shea E, . The cost effectiveness of the SIMPle intervention to improve antimicrobial prescribing for urinary tract infection in primary care. J Public Health (Oxf). 2017;39(4):e282-e289. 27679664 10.1093/pubmed/fdw102

[17] Gong CL, Zangwill KM, Hay JW, Meeker D, Doctor JN. Behavioral economics interventions to improve outpatient antibiotic prescribing for acute respiratory infections: a cost-effectiveness analysis. J Gen Intern Med. 2019;34(6):846-854. doi:10.1007/s11606-018-4467-x 29740788 PMC6544688

[18] Yoo M, Madaras-Kelly K, Nevers M, . A Veterans’ Healthcare Administration (VHA) antibiotic stewardship intervention to improve outpatient antibiotic use for acute respiratory infections: a cost-effectiveness analysis. Infect Control Hosp Epidemiol. 2022;43(10):1389-1395. doi:10.1017/ice.2021.393 34585655

[19] Zhang Z, Dawkins B, Hicks JP, . Cost-effectiveness analysis of a multi-dimensional intervention to reduce inappropriate antibiotic prescribing for children with upper respiratory tract infections in China. Trop Med Int Health. 2018;23(10):1092-1100. doi:10.1111/tmi.13132 30058210

[20] Mamun A, Zhao B, McCabe M, . Cost-benefit analysis of a population-based education program on the wise use of antibiotics. Can J Public Health. 2019;110(6):732-740. doi:10.17269/s41997-019-00245-w 31420845 PMC6964505

[21] Saqib K, Ivers N, Brown KA, . Spillover from an intervention on antibiotic prescribing for family physicians: a post hoc secondary analysis of a randomized clinical trial. JAMA Netw Open. 2025;8(7):e2518261. doi:10.1001/jamanetworkopen.2025.18261 40591360 PMC12215572

