# Supplementary material for: Mailed Audit and Feedback for Antibiotic Prescribing in Primary Care
Source: JAMA Netw Open. 2026 Mar 13;9(3):e261641. doi: 10.1001/jamanetworkopen.2026.1641 (PMC12988446; doi:10.1001/jamanetworkopen.2026.1641)
Supplement: Supplement 1. — eTable 1. List of Adverse Events, Undertreatment Harms, and Antibiotics eTable 2. Data Sources eFigure. Return on Investment by Number of Family Physicians [file jamanetwopen-e261641-s001.pdf]

Supplementary Online Content

Masucci L, Schwartz K, Ivers N, et al. Mailed audit and feedback for antibiotic prescribing in primary care. *JAMA Netw Open.* 2026;9(3):e261641. doi:10.1001/jamanetworkopen.2026.1641

eTable 1. List of Adverse Events, Undertreatment Harms, and Antibiotics
eTable 2. Data Sources
eFigure. Return on Investment by Number of Family Physicians

This supplementary material has been provided by the authors to give readers additional information about their work.

© 2026 Masucci L et al. *JAMA Network Open*.

eTable 1. List of Adverse Events, Undertreatment Harms, and Antibiotics

**Description**	**ICD Code**
**Adverse Events**	
Drug-induced autoimmune hemolytic anemia	D59.0
Drug-induced non-autoimmune haemolytic anaemia	D59.2
Drug-induced aplastic anemia	D61.1
Haemorrhagic disorder due to circulating anticoagulants	D68.3
Drug-induced headache, not elsewhere classified	G44.4
Drug-induced polyneuropathy	G62.0
Drug-induced myopathy	G72.0
Cardiomyopathy due to drugs and other external agents	I42.7
Hypotension due to drugs	I95.2
Toxic liver disease	K71
Drug-induced acute pancreatitis	K85.3
Generalized skin eruption due to drugs and medicaments	L27.0
Stevens-Johnson syndrome	L51.1
Toxic epidermal necrolysis	L51.2
Stevens-Johnson syndrome-toxic epidermal necrolysis overlap syndrome	L51.3
Drug-induced gout, multiple sites	M10.20
Drug-induced gout, upper arm	M10.22
Drug-induced gout, hand	M10.24
Drug-induced gout, pelvic region and thigh	M10.25
Drug-induced gout, lower leg	M10.26
Drug-induced gout, ankle and foot	M10.27
Drug-induced gout, other site	M10.28
Drug-induced gout, unspecified site	M10.29
Nephropathy induced by other drugs, medicaments and biological substances	N14.1
Nephropathy induced by unspecified drug, medicament or biological substance	N14.2
Anaphylactic shock due to adverse effect of correct drug or medicament properly administered	T88.6
Unspecified adverse effect of drug	T88.7
Drugs (antiviral) causing adverse effects in therapeutic use	Y41.5
Drugs (systemic anti-infective) causing adverse effects in therapeutic use	Y41.9
Observation for suspected toxic effect from ingested drug (includes adverse effect)	Z03.6
Urticaria	L50
Personal history of allergy to penicillin if no previous in last 2 years	Z88.0
Personal history of allergy to other antibiotic agents	Z88.1
Personal history of allergy to sulfonamides	Z88.2

© 2026 Masucci L et al. *JAMA Network Open*.

Personal history of allergy to other anti-infective agents	Z88.3
Renal failure	N17
Abdominal pain	R10
Nausea/vomiting	R11
Diarrhea	K52
Skin rash	R51
Adverse medication effect or allergy	*977
Rash	*691
Diarrhea	*009
Thrush	*112
**Enterocolitis due to *Clostridium difficile***	CDIFF
New antibiotic-resistant organisms (see ARO definition)	ARO
**Under Treatment Harms**	
ENT infections	J02.0 (confirmed Strep A pharyngitis), J03 (tonsillitis), H66.0 (AOM), H66.9 (AOM NOS), H70.0 (mastoiditis), J01.0-J01.9 (acute sinusitis)
SSTI	L03 (cellulitis and acute lymphadenitis), L03.0 – L.03.90 cellulitis of different parts of the body including unspecified cellulitis, ICD-10: L.01.0, L.01.1, L.08.0 (impetigo)
Invasive Infections	M72.6 (necrotizing fasciitis), A48.0 (gas gangrene), R65.2 (septic shock), A40.0 (sepsis), A40.1 (sepsis due to streptococcus, group B), A40.2 (sepsis due to Streptococcus, group D and enterococcus), A40.3 (sepsis due to streptococcal pneumonia), A40.8 (Other streptococcal sepsis), A40.9(streptococcal sepsis), A41.0-A41.9 (Other sepsis), P36.10 (sepsis of newborn due to unspecified streptococci) A48.3(streptococcal toxic shock syndrome), O85(puerperal sepsis), G00.0-G00.9 (bacterial meningitis), A39.0 (meningococcal meningitis), G06.0-G06.2 (brain/spine abscess), M00.0-M00.9 (pyogenic arthritis), L03.91, I89.1(lymphangitis), M86 (osteomyelitis), J86.0 (pyothorax with fistula), J86.9 (pyothorax without fistula), J18.9 & J15.4 (pneumonia), R78.81 (bacteraemia), A49.9 (bacteremia NOS),

© 2026 Masucci L et al. *JAMA Network Open*.

	Abscess (D73.3, E32.1, H60.0, H70.01, J36, J34.0, J39.x (x=0-2), J85, K11.3, K12.2, K61.x (x=0-4), K63.0, K65.1, K68.1, K75.0)), O41.12 P02.7 (chorioamnionitis), Endocarditis (I33.0, I33.9, I38, I39, I01.1), Endometritis (O86.12, O86.8, O86.89), Peritonitis (K65, N73.x (x=3-5), P78.1), Pericarditis (I01.0, I09.2, I30.x (x=1, 8, 9))
Rheumatic fever	I00 (rheumatic fever without heart involvement), I01(rheumatic fever with heart involvement), I02(rheumatic chorea)
**Antibiotics**	
Amoxicillin	
Amoxicillin & Clavulanic Acid Potassium	
Ampicillin	
Azithromycin	
Cefadroxil	
Cefixime	
Cefprozil	
Cefuroxime	
Cephalexin	
Ciprofloxacin HCL	
Clarithromycin	
Clindamycin	
Cloxacillin Sodium	
Doxycycline Hyclate	
Erythromycin	
Fosfomycin Tromethamine	
Levofloxacin	
Linezolid	
Minocycline HCL	
Moxifloxacin HCL	
Nitrofurantoin	
Norfloxacin	
Penicillin V Potassium	
Sulfamethoxazole & Trimethoprim	
Tetracycline HCL	
Trimethoprim	

© 2026 Masucci L et al. *JAMA Network Open*.

eTable 2. Data Sources

**ICES Databases**	**Description**
Ontario Drug Benefit Database	The ODB provides information on drugs administered in Ontario to patients eligible for publicly covered benefits (those <25 or >65 years of age or eligible for disability)
Discharge Abstract Database	The DAD includes information on all hospitalizations based on chart reviews including International Classification of Diseases-10 diagnosis codes and procedures performed during hospitalization.
Ontario Health Insurance Plan Claims Database	The OHIP databases contains all billing claims paid for by the Ontario Health Insurance Plan. Each record represents the delivery of service from a physician to a patient.
National Ambulatory Care Reporting System	The NACRS holds data on visits to healthcare institutions. This data includes demographics and the setting visited (day surgery, emergency department), and clinical data (e.g., diagnosis, treatment)
Complex Continuing Care Reporting System	The CCRS contains demographic, clinical, and resource utilization on individuals receiving continuing care services in hospitals or long-term care homes in Ontario, Canada.
Ontario Laboratory Information System	The OLIS contains information on all laboratory and diagnostic tests conducted.
National Rehabilitation Reporting System	The NRS holds data from participating adult inpatient rehabilitation programs.

© 2026 Masucci L et al. *JAMA Network Open*.

eFigure. Return on Investment by Number of Family Physicians

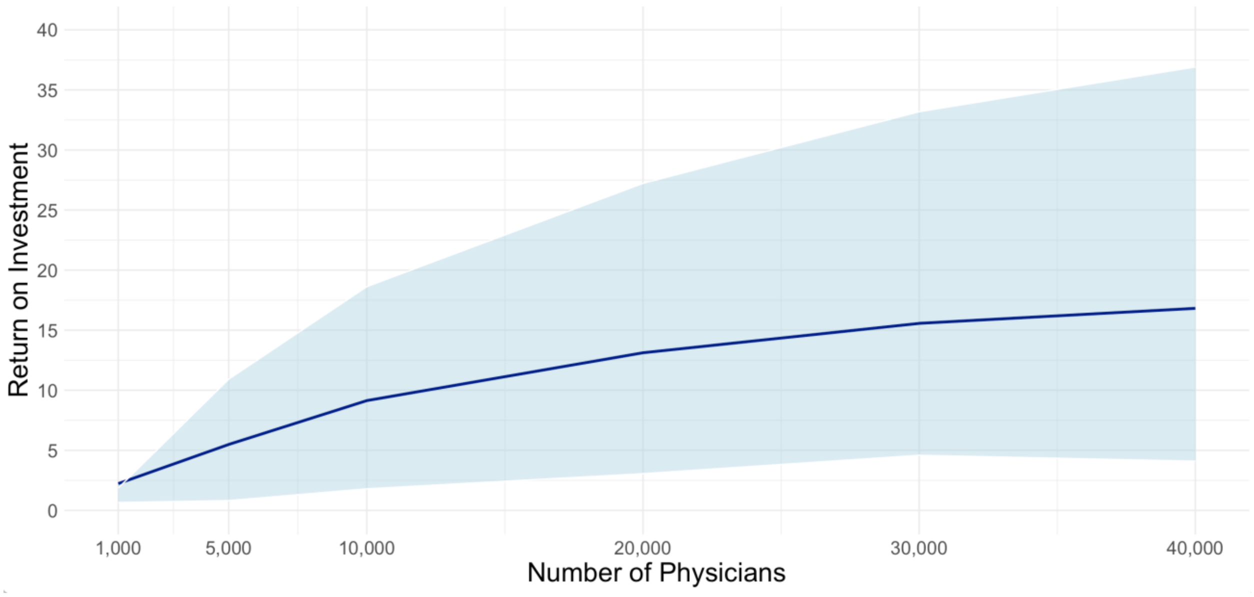

© 2026 Masucci L et al. *JAMA Network Open*.
