# Supplementary material for: Mailed Audit and Feedback for Antibiotic Prescribing in Primary Care
Source: JAMA Netw Open. 2026 Mar 13;9(3):e261641. doi: 10.1001/jamanetworkopen.2026.1641 (PMC12988446; doi:10.1001/jamanetworkopen.2026.1641)
Supplement: Supplement 2. — Data Sharing Statement [file jamanetwopen-e261641-s002.pdf]

Data Sharing Statement

Masucci. Mailed Audit and Feedback for Antibiotic Prescribing in Primary Care. *JAMA Netw Open*. Published March 13, 2026. doi:10.1001/jamanetworkopen.2026.1641

Data
Data available: Yes
Data types: Other (please specify)
Additional Information: The dataset from this study is held securely in coded form at ICES. While legal data sharing agreements between ICES and data providers (eg, healthcare organizations and government) prohibit ICES from making the dataset publicly available, access may be granted to those who meet pre-specified criteria for confidential access, available at www.ices.on.ca/DAS (email: das@ices.on.ca). The full dataset creation plan and underlying analytic code are available from the authors upon request, understanding that the computer programs may rely upon coding templates or macros that are unique to ICES and are therefore either inaccessible or may require modification.
How to access data: The full dataset creation plan and underlying analytic code are available from the authors upon request at kthavorn@ohri.ca
When available: With publication

Supporting Documents
Document types: Statistical/analytic code
How to access documents: These documents may be accessed through the corresponding author.
When available: With publication

Additional Information
Who can access the data: Researchers whose proposed use of the dataset creation plan and analytic code has been approved by the authors.
Types of analyses: To conduct similar research on antimicrobial resistance/prescribing.
Mechanisms of data availability: After approval of a proposal
